# Transient Early Fine Motor Abnormalities in Infants Born to COVID-19 Mothers Are Associated With Placental Hypoxia and Ischemia

**DOI:** 10.3389/fped.2021.793561

**Published:** 2022-01-06

**Authors:** Huan-Yu Liu, Juanjuan Guo, Chang Zeng, Yuming Cao, Ruoxi Ran, Tiancheng Wu, Guifang Yang, Dongchi Zhao, Pu Yang, Xuechen Yu, Wei Zhang, Song-Mei Liu, Yuanzhen Zhang

**Affiliations:** ^1^Department of Gynaecology and Obstetrics, Zhongnan Hospital of Wuhan University, Wuhan, China; ^2^Hubei Clinical Research Center for Prenatal Diagnosis and Birth Health, Wuhan, China; ^3^Department of Preventive Medicine, Northwestern University Feinberg School of Medicine, Chicago, IL, United States; ^4^Department of Clinical Laboratory, Center for Gene Diagnosis, and Program of Clinical Laboratory, Zhongnan Hospital of Wuhan University, Wuhan, China; ^5^Department of Pathology, Zhongnan Hospital of Wuhan University, Wuhan, China; ^6^Department of Pediatrics, Zhongnan Hospital of Wuhan University, Wuhan, China; ^7^Institute of Precision Medicine, Jining Medical University, Jining, China

**Keywords:** COVID-19, fine motor skills, placental mtDNA, infants, physical and neurobehavioral development

## Abstract

**Background:** Long-term effects of Coronavirus Disease 2019 (COVID-19) on infants born to infected mothers are not clear. Fine motor skills are crucial for the development of infant emotional regulation, learning ability and social skills.

**Methods:** Clinical information of 100 infants born to 98 mothers (COVID-19 *n* = 31, non-COVID-19 *n* = 67) were collected. Infants were follow-up up to 9 months post-partum. The placental tissues were examined for SARS-CoV-2 infection, pathological changes, cytokines, and mtDNA content.

**Results:** Decreased placental oxygen and nutrient transport capacity were found in infected pregnant women. Increased IL-2, IL-6, TNF-α, and IFN-γ were detected in trophoblast cells and maternal blood of COVID-19 placentas. Elevated early fine motor abnormal-ities and increased serum TNI (troponin I) levels at delivery were observed in infants born to mothers with COVID-19. Increased abnormal mitochondria and elevated mtDNA content were found in the placentas from infected mothers. The placental mtDNA content of three infants with abnormal DDST were increased by 4, 7, and 10%, respectively, compared to the mean of the COVID-19 group. The Maternal Vascular Malperfusion (MVM), elevated cytokines and increased placental mtDNA content in mothers with COVID-19 might be associated with transient early fine motor abnormalities in infants. These abnormalities are only temporary, and they could be corrected by daily training.

**Conclusions:** Babies born to COVID-19 mothers with mild symptoms appeared to have little or no excess long-term risks of abnormal physical and neurobehavioral development as compared with the infants delivered by non-COVID-19 mothers.

## Introduction

Coronavirus Disease 2019 (COVID-19) is a respiratory tract infection caused by severe acute respiratory syndrome coronavirus 2 (SARS-CoV-2) and has resulted in a global pandemic (1). A number of studies have described the clinical characteristics and outcomes of COVID-19 pregnancies and their babies (2). In the United States alone, over 84,000 pregnant women have been infected with SARS-CoV-2 as of April 12th, 2021 (3). Our previous observational study suggested that there was no evidence for intrauterine infection caused by vertical transmission in COVID-19 women in late pregnancy at the beginning of the pandemic in China (4). To date, no serious adverse outcomes have been reported in neonates born to SARS-CoV-2-positive mothers (2). Some reports in both adults and children have associated COVID-19 with a variety of central and peripheral neurological insults (5).

Whether gestational exposure to SARS-CoV-2 infection could have a long-term impact on postnatal growth and neurobehavioral development has not been investigated. Of particular interest to us is fine motor development, which is an important component for children general growth and successful participation in daily activities. Children with a history of transient neurological abnormalities are at a higher risk for poorer cognitive and academic skills than those with normal neurological findings during their 1st year of school (6). Transient neurodevelopmental abnormalities in infancy are also associated with developmental abnormalities at 2–3 years of age and learning abnormalities at 5 years of age (7). Calame et al. also found an increased rate of delayed language development and behavioral abnormalities at age 3 in children with transient neurologic abnormalities (8). These results suggested that long-term impact on infants' neurodevelopment after COVID-19 needs to be investigated with more cases, multidimensional indicators and longer time follow-up. Previous studies have found that infants manifesting fine motor problems could benefit from early intervention to improve their development and to prevent further complications (9–12). Therefore, it is of great importance to monitor fine motor development in infants born to COVID-19 mothers that may provide urgently needed information for possible early intervention in affected children.

Physiologically, the placenta serves a myriad of functions that support fetal growth and survival (13). In the cell, mitochondria play a central role in maintaining oxidative homeostasis and reactive oxide species (ROS) production (14). Restricted nutrients, oxidative stress, and inflammation may impair mitochondrial oxidative phosphorylation, leading to reduced ATP and excessive ROS (15, 16). Since the newborn brain accounts for almost 60% of total body oxygen and glucose consumption (17), this level of high-energy demand makes brain development particularly susceptible to impairment from nutritional shortfalls (18). Moreover, brain glucose requirement is associated inversely to body growth from infancy to puberty (18). To compensate for defective mitochondrial functions, the placenta could undergo metabolic adaptations by promoting mitochondrial biogenesis (15). As a matter of fact, elevated mtDNA copy numbers in the placentas have been associated with reduced birthweight, suggesting that mtDNA might be a potential biomarker for placental insufficiency (19, 20).

With the on-going global pandemic, the impact of COVID-19 on pregnant women, newborns have received worldwide attention. The Maternal Vascular Malperfusion (MVM) was found in placentas of mothers with COVID-19 (21–23). Under the MVM, whether there is mitochondrial dysfunction in the placentas of pregnant women with COVID-19 and whether these abnormalities are associated with developmental outcomes in infants remain unclear. Specifically, in the current study, taking advantage of our clinical resources, we explored the placental pathology and mitochondrial function of COVID-19 in pregnancy, examined the developmental outcomes of affected infants after the follow-up of ~9 months, and evaluated their correlations.

## Materials and Methods

### Study Design and Participants

**Figure 1:**
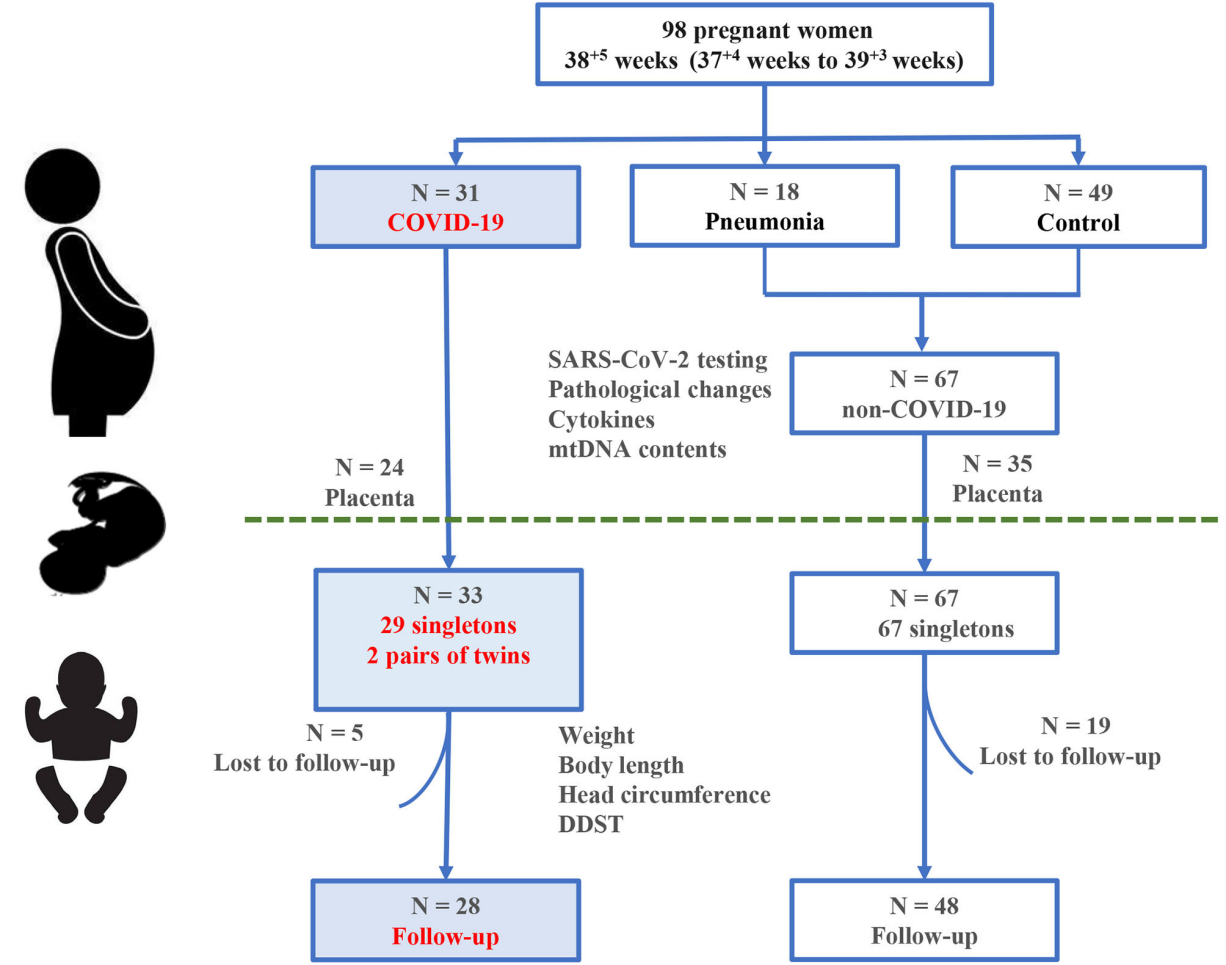
Study design and participants. A total of 98 pregnant women and 100 infants born to them are included in this study.

The study design is shown in Figure 1. A total of 98 pregnant women (COVID-19, *n* = 31; non-COVID-19 pneumonia, *n* = 18; controls, *n* = 49) and 100 newborns were recruited at Zhongnan Hospital of Wuhan University, China during 01/2020-1/2021. SARS-CoV-2 infection was confirmed with both RT-PCR test and serum antibody assay according to the Chinese Novel Coronavirus Pneumonia Prevention and Control Program (7th edition) (24). Among the 18 non-COVID-19 pregnancies with pneumonia, there were 2 cases of influenza A infection, 4 cases of both influenza A and B infections, 5 cases of bacterial infections, the rest 7 cases had no pathogenic confirmation. The 49 controls were pregnant women matched for age, gestational age at delivery (GAD), and underlying illnesses including pregnancy-induced hypertension (PIH) and gestational diabetes mellitus (GDM). All pregnant women delivered by cesarean section and newborns were transferred to the Neonatal Intensive Care Unit (NICU) to reduce the risk of SARS-CoV-2 infection (4). This study was reviewed and approved by the Medical Ethical Committee of Zhongnan Hospital of Wuhan University (Approval Number: 2020080H).

### Clinical Data Collection

Demographic and clinical information were collected from medical records. Laboratory testing included routine blood cell counts, coagulation function, liver function, renal function, serum electrolytes, myocardial markers, lipids, inflammatory markers and cytokines.

Body weight and length are the most important and sensitive indicators that reflect children's growth and development, while the head circumference reflects the degree of development of the brain and skull. Therefore, weight, body length and head circumference were measured by a neonatologist. The standard growth curve was plotted according to the 2009 reference standards from the Ministry of Health of China (25). The development of a premature infant was evaluated with corrected age. The Denver Developmental Screening Test (DDST, 2nd Edition) (26) was used to evaluate neurodevelopmental levels for all infants in this study.

### Parent-Guided Postnatal Training and Intervention

To improve fine motor performance for infants with fine motor skills abnormalties by the DDST, parents were advised to train their babies for at least 5 months, including visual motor perception (e.g., picking up the rattle) and fine motor training-based intervention (e.g., throwing, catching, opening hands, touching, grasping, and bimanual task). The frequency was 5–10 min once for 3–5 times per day (9, 27). Additionally, the infants were asked to be breast-feeding and lived in the enriched environments (28, 29).

### Placental Characterization and Determination of mtDNA Copy Numbers

The pathologies of placentas were examined according to the Amsterdam Placental Workshop Group Consensus Statement (30). The MVM includes pathological features such as placental infarction, decidual vascular disease, accelerated villous maturation, and villous infarction (30). We collected 10 placentas from COVID-19 pregnant women with infection duration ≥7 days and 10 placentas from matched non-COVID-19 pregnant women as described in Study Design and Participants. Supplementary Table S7 summarizes their clinical and pathological findings. The placental tissues were examined for SARS-CoV-2 infection using the RT-PCR with the Daan Gene Kit (Guangzhou, China).

The H&E staining was used to observe the pathological features of placentas, following the standard protocol (31). Placental interhemal membrane (IHM) is a trilaminar barrier that is made up of syncytiotrophoblast, cytotrophoblast and fetal capillary endodermis cells. Thicker IHM may hamper transport efficiency of nutrients especially oxygen. Even slight thickening of the placental IHM may reduce the placental oxygen diffusion capacity significantly (32). The average thickness of the placental IHM was measured along the direction of the test line, from intersections between the test line and the fetal capillaries to the closest maternal blood space through the orthogonal intercepts method (33).

Cytokine levels in the placental tissues were performed by the Enzyme Linked Immunosorbent Assay (ELISA) with commercial ELISA kits (Beijing 4A Biotech Co., Ltd) according to the instructions. The concentrations of cytokines were calculated according to standard curves by a special program for the evaluation of ELISA results. Transmission electron microscopy (Hitachi TEM system) was used to detected mitochondrial morphology. The qPCR that simultaneously targeted a single-copy nuclear gene (β_2_-microglobulin, β_2_M) and the tRNA Leu (UUR) gene in mtDNA was used to quantify mtDNA copy numbers (34).

### Statistical Analysis

All statistical analyses were performed using the SPSS 21.0 (SPSS Inc., Chicago, IL, USA), or under the R Statistical Computing Environment (v 3.6.2). The one-way ANOVA or the Student's *t*-test were used to compare the differences in normally distributed data. The Mann-Whitney-test was used to assess the differences in skewed data. The Chi-square-test was conducted to test the categorical variables. Linear mixed-effect models with fixed mean and random intercept were implemented to model the within-subject data and determine whether age, sex, and COVID-19 infection status were able to predict the physical development of infants. *P*-value < 0.05 was used for statistical significance. The Bonferroni correction was used for evaluating multiple comparisons.

## Results

### Baseline Clinical Features of Mothers

Table 1 summarizes the clinical characteristics of the pregnancies (*n* = 98). The mean age, BMI, GAA, gravidity and parity did not differ across the three groups (COVID-19, *n* = 31; non-COVID-19 pneumonia, *n* = 18; controls, *n* = 49). The baseline medical conditions of the COVID-19 pregnancies included PIH (19.4%), GDM (12.9%), cardiovascular diseases (3.2%), liver dysfunction (3.2%), hepatitis B virus carrier (16.1%), hepatitis E virus carrier (3.2%), and influenza infection (9.7%). No significant difference in the comorbidities was found across different groups, except for hepatitis B virus carrier and influenza infection. Of note, main clinical manifestations of the COVID-19 pregnancies included fever (58.1%), cough (41.9%), muscle soreness (9.7%), dyspnea (1.4%), and diarrhea (1.4%), and were similar to a previous study (35). All of the pregnant women were moderate disease. Only 22 cases (71.0 %) had typical symptoms of SARS-COV-2 infection, the rest of 9 patients (29.0%) showed typical images of multiple patchy ground-glass shadows in lungs.

**Table 1:** Baseline characteristics and clinical features of pregnant women.

**Variable**	**COVID-19**	**Pneumonia**	**Control**	***P* ^a^**
	**(*N* = 31)**	**(*N* = 18)**	**(*N* = 49)**	
Age, years	30.5 ± 3.4	32.3 ± 4.3	31.7 ± 4.6	0.26
BMI, kg/m^2^	29.93 ± 0.38	30.01 ± 0.43	28.75 ± 0.65	0.18
SBP, mmHg	126.1 ± 3.2	116.3 ± 1.8	117.0 ± 3.2	0.04
DBP, mmHg	82.8 ± 2.4	75.2 ± 1.8	76.6 ± 1.9	0.04
GAA, weeks	38.0 ± 0.4	38.7 ± 0.5	38.2 ± 0.3	0.54
Gravidity	2 (1, 2)	1 (1, 3)	1 (1, 2)	0.70
Parity	0 (0, 1)	0 (0, 1)	0 (0, 1)	0.80
Twin pregnancy^b^	2 (6.5)	0 (0)	1 (2.0)	0.58
**Comorbidities**				
PIH^b^	6 (19.4)	0 (0)	10 (20.4)	0.10
GDM^b^	4 (12.9)	3 (16.7)	5 (10.2)	0.72
Cardiovascular disease^b^	1 (3.2)	0 (0)	0 (0)	0.50
Liver dysfunction^b^	1 (3.2)	0 (0)	0 (0)	0.50
Hypothyroidism^b^	0 (0)	0 (0)	2 (4.1)	0.68
Hepatitis B virus carrier^b^	5 (16.1)	2 (11.1)	0 (0)	0.008
Hepatitis E virus carrier^b^	1 (3.2)	0 (0)	0 (0)	0.50
Influenza infection^b^	3 (9.7)	6 (33.3)	0 (0)	0.0001

a *P-values were derived from one-way ANOVA analysis (quantitative variables) or chi-square test (categorical variables), indicating differences across COVID-19, non-COVID-19 pneumonia and control pregnant women*.

b *Data are expressed as percentage*.

*P < 0.05 was considered statistically significant*.

*BMI, Body Mass Index; GAA, Gestational age on admission; SBP, systolic blood pressure; DBP, diastolic blood pressure; PIH, Pregnancy-induced hypertension; GDM, Gestational diabetes mellitus*.

Laboratory findings were available from all of the 98 pregnant women (Supplementary Table S1). Elevated myocardial enzymes, severe coagulopathy, and abnormal liver function were common features of the COVID-19 pregnant women (Supplementary Table S1). In particular, serum lactate dehydrogenase (LDH) was significantly elevated in the COVID-19 group relative to the women with non-COVID-19 pneumonia. Additionally, the incidence of abnormal LDH in the COVID-19 group (7/25) was higher compared to those with non-COVID-19 pneumonia (0/19) (*P* = 0.01; Supplementary Table S2).

### Baseline Clinical Features of Newborns

We combined the controls and those infants from those mothers with non-COVID-19 pneumonia into a single “non-COVID-19” group, because their clinical features appeared to be comparable (Table 2). All 100 live births (58 male and 42 female neonates), among whom 33 babies (29 singletons and 2 pairs of twins) were born to 31 women with COVID-19 (Table 2). After removing the neonates from mothers with PIH and GDM, the average birthweight of newborns in the COVID-19 group was significantly lower than those infants in the non-COVID-19 group (3.03 ± 0.13 kg vs. 3.29 ± 0.06 kg, *P* = 0.03). All neonates in the COVID-19 group were tested negative for SARS-CoV-2. In addition, all babies showed negative results when they underwent serum SARS-CoV-2 antibody assay at the age of 1 month. Chest X-ray was performed in 25 newborns born to COVID-19 mothers who were immediately transferred to the NICU after birth. One infant born to a COVID-19 mother had patchy shadow in the middle lobe of right lung in the first exam, but negative in the replicate tests.

**Table 2:** Baseline characteristics and clinical characteristics of the follow-up infants.

**Variable**	**COVID-19**	**Non-COVID-19**	***P* ^a^**
	**(*N* = 33)**	**(*N* = 67)**	
Sex (Male)^b^	20 (60.6)	38 (56.7)	0.71
GAD, weeks	38.0 ± 0.4	37.8 ± 0.7	0.82
Birthweight, kg	2.99 ± 1.13	3.15 ± 0.08	0.25
Body length, cm	48.35 ± 0.88	49.20 ± 0.49	0.37
Head circumference, cm	33.74 ± 0.37	33.60 ± 0.22	0.74
Low birth weight^b^	5 (15.2)	10 (14.9)	>0.99
Premature delivery^b^	6 (18.2)	16 (23.9)	0.52

a *P-values were derived from student's test (quantitative variables) or chi-square test (categorical variables), indicating differences across COVID-19, non-COVID-19 Pneumonia and Control pregnant women*.

b *Data are expressed as percentage*.

*P < 0.05 was considered statistically significant*.

*GAD, Gestational age at delivery*.

Overall, the laboratory testing results of the COVID-19 group had a significantly lower levels of white blood cells (*P* = 0.02), lymphocytes (*P* = 0.005), and aminotransferase (*P* = 0.03). Notably, the median of serum cardiac troponin I (TNI) showed ~2-fold increase in newborns in the COVID-19 group (20.32 ± 3.41 pg/mL vs. 9.66 ± 1.27 pg/mL, *P* = 0.007) (Supplementary Table S3), reflecting a strikingly different rate of abnormal TNI (20%, 5/25) in the COVID-19 group vs. (0%, 0/23) in the non-COVID-19 group (*P* = 0.05; Supplementary Table S4). Overall, the average birthweight of newborns was significantly decreased in the COVID-19 group when excluding maternal complications.

### Development Outcomes in Infants

In total, 76 infants (COVID-19, *n* = 28; non-COVID-19, *n* = 48, including 11 infants born to mothers with non-COVID-19 pneumonia mothers) were followed up between March and November, 2020, with the median follow-up of 5 months. The main reason for loss of follow-up was parental decline to further participate in the study.

No significant differences in clinical and developmental parameters were found between the two groups, including sex, GAD, birthweight, body length and head circumference at birth. The proportion of preterm showed no difference between the two groups (17.9 vs. 14.3%) (Supplementary Table S5).

**Figure 2:**
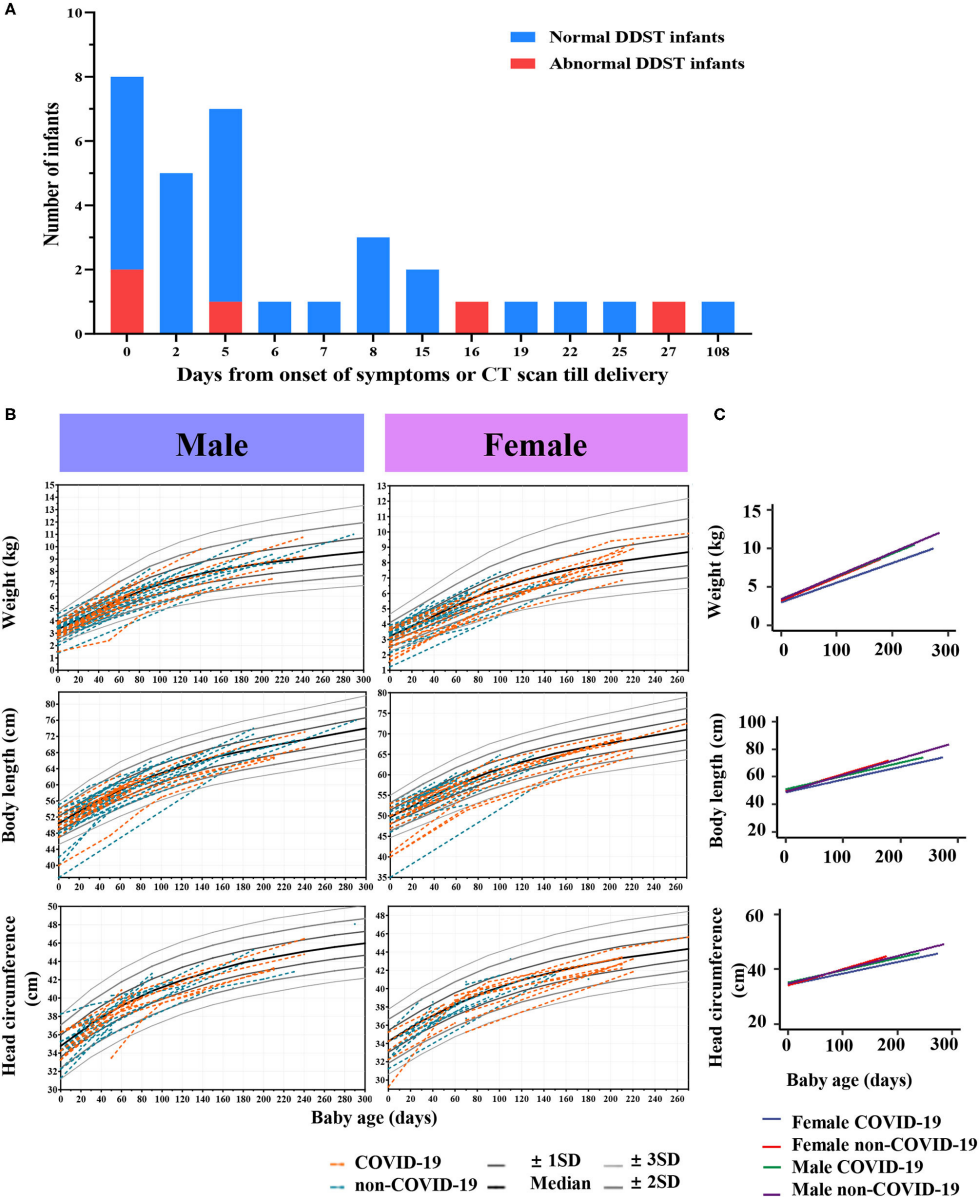
Follow-up outcomes of infants. **(A)** Distributions of infants with normal or abnormal DDST delivered by COVID-19 mothers. **(B)** Infants' growth curves of weight, body length and head circumference in the COVID-19 group and the non-COVID-19 group. **(C)** Linear mixed-effect models with fixed mean and random intercept were developed to model the within-subject data and determine whether age, sex, and COVID-19 infection status were able to predict the physical development of infants.

The Physical Development Index (i.e., weight, body length, and head circumference) of infants were collected and compared. Table 3 presents results from a linear mixed-effect model predicting physical development of infants. The growth curve of weight, body length and head circumference showed a similar trend in the COVID-19 group compared to the non-COVID-19 group (*P* > 0.05; Figures 2B,C). In female infants, the COVID-19 group had 0.45 kg lower weight, 2.44 cm shorter in body length and 1.01 cm smaller in head circumference as compared with the non-COVID-19 group (All *P* < 0.05). In contrast, there was no significant difference in physical development between the two groups among male infants (Table 3).

**Table 3:** Linear mixed-effect models predicting physical development of infants.

**Variable**	**Weight, kg**			**Body length, cm**			**Head circumference, cm**		
	**β**	**95%CI**	***P* ^a^**	**β**	**95%CI**	***P* ^a^**	**β**	**95%CI**	***P* ^a^**
**All**									
Non-COVID-19	Ref			Ref			Ref		
COVID-19	−0.23	−0.53, 0.07	0.14	−1.03	−2.69, 0.63	0.23	−0.43	−1.01, 0.15	0.15
**Male**									
Non-COVID-19	Ref			Ref			Ref		
COVID-19	−0.01	−0.43, 0.41	0.95	0.14	−2.37, 2.65	0.91	0.05	−0.71, 0.81	0.89
**Female**									
Non-COVID-19	Ref			Ref			Ref		
COVID-19	−0.45	−0.88, −0.02	0.05	−2.44	−4.42, −0.46	0.02	−1.01	−1.92, −0.10	0.04

a *P-values were derived from student's test indicating differences between infants of COVID-19 and non-COVID-19 pregnant women*.

*P < 0.05 was considered statistically significant*.

*GAD, Gestational age at delivery; The linear mixed-effect models with fixed mean and random intercept were developed to model the within-subject data and determine whether COVID-19 infection status was able to predict the physical development of infants, including weight, body length, and head circumference. CI, confidence interval*.

We observed fine motor abnormalities in five infants born to mothers with COVID-19, significantly higher than in the control group (15.2%, 5/28 vs. 2.1%, 1/48, *P* = 0.02) (Figure 2A). The maternal-infant demographic and clinical findings in infants with abnormal DDST are listed in Supplementary Table S6. The 5 infants in the COVID-19 group showing neurodevelopmental delay were full-term, normal in birthweight, and their mothers had no complications such as PIH or GDM. Notably, these 5 infants showed delay in “Fine motor-adaptive” domain at the age of 1–2 months (Table 4). Parents of these infants were asked to perform parent-guided postnatal training and intervention. Specifically, case 9 and case 11 passed the examinations in the second DDST at the age of 6–7 months. Additionally, case 3, 9, and 11 passed examinations at the age of 12–13 months, indicating that the observed neurodevelopmental delay would be short-term effects that could be corrected by postnatal training.

**Table 4:** Demographic and clinical findings in the 5 infants in the COVID-19 group with a diagnosis of neurodevelopmental delay by the DDST.

**Case**	**Sex**	**Age (days)**	**GAD (weeks)**	**Birth weight (kg)**	**DDST**					
					**Testing age (months)**	**Personal-social**	**Fine motor**	**Gross motor**	**Language**	**Rated**
										
2	Female	46	38^+2^	3.79	1	Pass	**Caution**	Pass	Pass	**Questionable**
3	Female	63	37^+4^	2.63	2	Pass	**Caution**	Pass	Pass	**Questionable**
9	Female	65	38	5.06	2	Pass	**Caution**	Pass	Pass	**Questionable**
		210			7	Pass	Pass	Pass	Pass	Normal
11	Male	69	37^+1^	2.81	2	Pass	**Caution**	Pass	Pass	**Questionable**
22	Male	63	39^+3^	2.57	2	Pass	**Caution**	Pass	Pass	**Questionable**
		208			6	Pass	Pass	Pass	Pass	Normal

*DDST, Denver Developmental Screening Test; GAD, Gestational age at delivery*.

*The bold fonts indicate the information of abnormal infants. “Pass” indicates the child is able to do the task. “Caution” indicates the child is not able to do the task that 75% of his/her age matched children can do. If the infant has 1 “Caution,” the rated results would be considered as “Questionable”*.

### Maternal Vascular Malperfusion Were Present in COVID-19 Placentas

**Figure 3:**
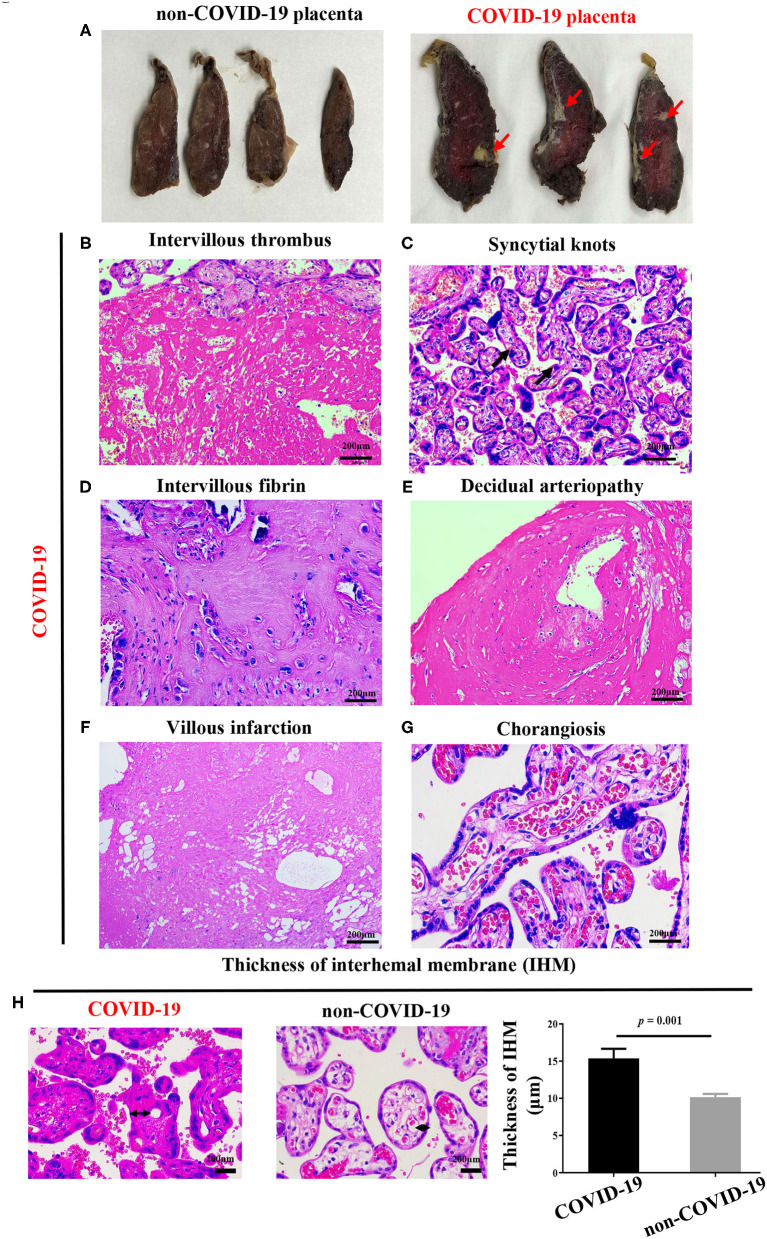
Pathological changes of COVID-19 placentas. **(A)** Sagittal plane of COVID-19 placentas showing hypoxia and infarction, **(B–H)** H&E stains of COVID-19 placentas showing **(B)** intervillous thrombus **(C)** syncytial knots and **(D)** intervillous fibrin, **(E)** decidual arteriopathy, **(F)** villous infarction, **(G)** chorangiosis, and **(H)** increased thickness of IHM, as compared with non-COVID-19 placentas, indicating placental oxygen and nutrient transport capacity are decreased. *P-*values were derived from the Student's *t-*test.

We observed that more than 5% of placental infarcts were observed in the sagittal surface of the COVID-19 placentas (Figure 3A). The rate of increased intervillous fibrin was higher in the COVID-19 group (7/10 vs. 0/10, *P* = 0.03) (Figure 3D, Supplementary Table S8). Besides, increased intervillous thrombus (4/10, Figure 3B), syncytial knots (5/10, Figure 3C), decidual arteriopathy (6/10, Figure 3E) and villous infarction (6/10, Figure 3F) were also observed in COVID-19 pregnancies, but showed no statistical differences between the two groups (Supplementary Table S8). Additionally, we also observed chorangiosis in COVID-19 placentas (Figure 3G). Of note, we found that the placental IHM thickness significantly increased in the COVID-19 group (*P* = 0.001, Figure 3H). These results supported that hypoxia and ischemia were present in COVID-19 placentas.

### Increased Cytokines in COVID-19 Placentas

**Figure 4:**
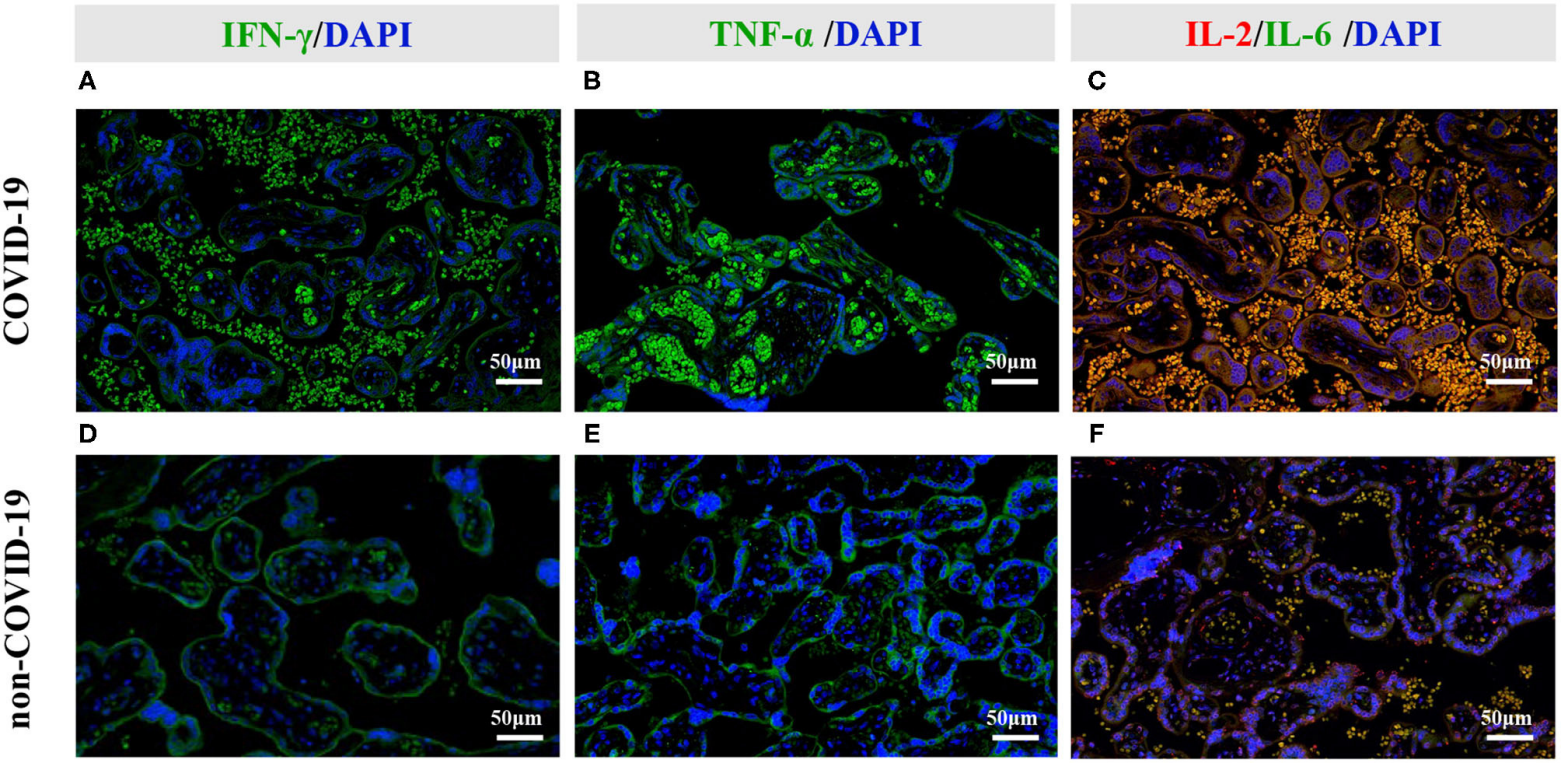
Representative photomicrographs of the immunofluorescent staining of IL-2, IL-6, IFN- γ, and TNF-α in the placental tissues. **(A–C)** Immunofluorescent staining of COVID-19 placentas. **(D–F)** Immunofluorescent staining of non-COVID-19 placentas. **(A,D)** Increased IFN-γ signal was primarily co-localized with maternal blood in COVID-19 placentas. **(B,E)** Increased TNF-α signal was primarily co-localized with placental trophoblast cells in COVID-19 placentas. **(C,F)** Increased IL-2 and IL-6 signals were primarily co-localized with the maternal blood in COVID-19 placentas.

Because placental ischemia also stimulates the release of cytokines, we further explored the level and location of cytokines. Compared with that in the non-COVID-19 group, the levels of Interleukin-2 (IL-2), Interleukin-6 (IL-6), Interferon-gamma (IFN-γ) and tumor necrosis factor-alpha (TNF-α) were significantly increased in the COVID-19 group (*P* < 0.05; Table 5). Immunofluorescent staining further revealed that IFN-γ was co-localized with the maternal blood in the placentas (Figures 4A,D), but TNF-α was primarily co-localized with placental trophoblast cells in the COVID-19 group (Figures 4B,E). In addition, our immunofluorescent staining also showed co-localization of IL-2 with IL-6 was primarily in maternal blood in COVID-19 placentas (Figures 4C,F). These results indicated that hypoxia and ischemia in COVID-19 placentas could promote cytokines production by placental trophoblast cells.

**Table 5:** Levels of cytokines in the placental tissues.

**Cytokine**	**COVID-19**	**Non-COVID-19**	***P* ^a^**
	**(*N* = 24)**	**(*N* = 25)**	
IFN-α, pg/mL	5.93 ± 0.67	9.14 ± 1.68	0.06
IFN-β, pg/mL	3.19 ± 2.08	4.30 ± 1.27	0.69
IFN-γ, pg/mL	25.50 ± 4.95	9.03 ± 2.04	0.006
IL-1β, pg/mL	8.97 ± 1.84	8.08 ± 1.49	0.73
IL-2, pg/mL	154.63 ± 1.95	144.79 ± 3.82	0.02
IL-4, pg/mL	2.18 ± 0.51	2.12 ± 0.89	0.95
IL-6, pg/mL	67.41 ± 6.26	48.01 ± 5.72	0.04
IL-7, pg/mL	6.52 ± 0.24	6.61 ± 0.22	0.79
IL-10, pg/mL	8.02 ± 1.25	4.18 ± 0.54	0.02
TNF-α, pg/mL	49.50 ± 0.94	45.17 ± 0.60	0.001

a *P-values were derived from the Student's t-test. P < 0.05 was considered statistically significant*.

*IFN-α, interferon alpha; IFN-β, interferon Beta; IFN-γ, interferon gamma; IL, interleukin; TNF-α, tum-or necrosis factor α*.

### Increase of Abnormal Mitochondria and mtDNA Content

**Figure 5:**
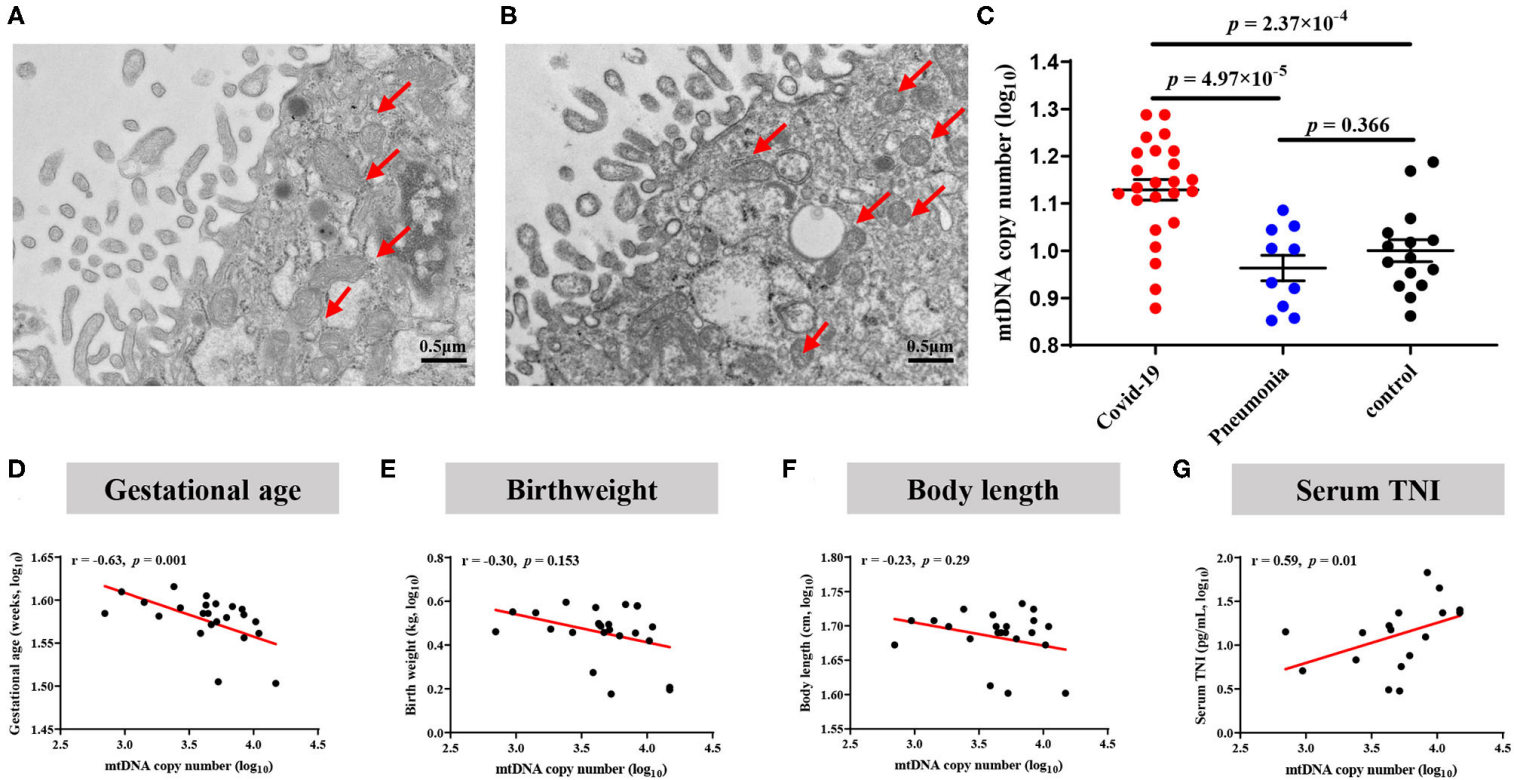
Alterations of mitochondrial morphology and mtDNA copy numbers in placentas. **(A)** Normal mitochondrial morphology in healthy placentas. **(B)** Elevated abnormal mitochondria in COVID-19 placentas. **(C)** Comparison of placental mtDNA copy numbers across COVID-19, pneumonia and Control group. *P*-values were derived from the Student's *t-*test. **(D–G)** COVID-19 pregnant women with increased placental mtDNA were associated with decreased birthweight. **(D)** Association of placental mtDNA and gestational age. **(E)** Association between placental mtDNA and birthweight. **(F)** Association between placental mtDNA and body length. **(G)** Association between placental mtDNA and neonatal serum TNI.

As shown in Figures 5A–D, the proportion of abnormal mitochondria was increased in the villous syncytiotrophoblast of COVID-19 pregnant, indicating that mitochondrial dysfunction likely occurred in the placentas from infected women. The placental mtDNA content was also found to be elevated in COVID-19 mothers, as compared with the non-COVID-19 controls (4.66 ± 0.07 vs. 4.19 ± 0.06, *P* = 6 × 10^−5^) (Figure 5C).

### Association Between Placental mtDNA Content and Early Physical and Neurobehavioral Development

To investigate whether the increase of placental mtDNA content is associated with offspring health, we explored the correlation between placental mtDNA content and offspring outcome. We only found that the copy number of placental mtDNA was associated with GAD (*r* = −0.65, *P* = 0.001) and TNI (*r* = 0.59, *P* = 0.01) in the COVID-19 group (Figures 5D–G). Three infants with abnormal DDST results demonstrated placental mtDNA content increasing by 4, 7, and 10%, respectively, compared to the mean of the COVID-19 group.

## Discussion

In the current study, we reported that poor maternal vascular perfusion, elevated cytokines production, increased abnormal mitochondria and elevated mtDNA copy numbers in the placentas of COVID-19 mothers. Early fine motor abnormalities which could be corrected in 6–7 months were elevated in the COVID-19 group compared to the non-COVID-19 group, supporting that neurodevelopmental delay might be short-term effects. Nevertheless, a similar trend of growth curve was shown in the COVID-19 and non-COVID-19 infants in the follow-up, indicating limited effects of COVID-19 on growth parameters after birth. In addition, mtDNA copy numbers appeared to be negatively associated with birthweight and GAD, and positively associated with infant serum TNI. Notably, infants with higher mtDNA content in placentas had a higher abnormal rate for the DDST at 1–2 months of age.

Significantly, poor MVM was the most significant feature associated with COVID-19 in the current study. Moreover, placental IHM thickening was also found in COVID-19 pregnancies that could influence placental oxygen and nutrient transport capacity. The MVM and chronic histiocytic intervillositis with trophoblast necrosis were found in COVID-19 placentas in several case reports (21, 22, 36, 37). Taken together, our findings provided support for the MVM in placentas from COVID-19 mothers, which could directly affect the fetal development *in utero* (38, 39).

It is also established that placental hypoxia may alter mitochondrial functions and mtDNA content, which eventually lead to fetal growth restriction (40). It is not clear whether mitochondrial functions and placental mtDNA would be changed in COVID-19 pregnant women, and their potential association with developmental delay. In agreement with previous reports, we found that the proportion of abnormal mitochondria was elevated in COVID-19 placentas. It is reasoned that to compensate for defective mitochondrial functions, their copy numbers would increase to fulfill energy demand for pregnancy maintenance under the placental hypoxia and ischemia status (15).

The so-called “cytokine storm” was observed in severe COVID-19 cases. Specifically, IFN-γ, IL-6, TNF-α, IL-10, IL-1, IL-5, and IL-8 were the main mediators behind cytokine storm (41, 42). Thus, evaluation of cytokine profiles in COVID-19 placentas attracted our attention. In severe COVID-19 cases, viral proteins may induce pro-inflammatory cytokines (43, 44). It is reported that IL-2, IL-6, TNF-α, and IFN-γ are increased in peripheral blood of COVID-19 pregnant and the level of IFN-γ is positively correlated with disease severity in pregnant women with COVID-19 (45). In the COVID-19 placentas, some studies observed elevated IL-5 levels as well as decreased IL-7 and TNF-related apoptosis-inducing ligand levels. In particular, the level of IFN-γ was higher in placentas that tested positive for both viral nucleic acids and proteins relative to placentas that tested positive only for the viral proteins (46). However, significantly higher levels of IL-2, IL-6, TNF-α, and IFN-γ were found in the pregnant women with COVID-19 infection. The locations of these cytokines were primarily in trophoblast cells and the maternal blood in placentas. Of note, under the placental hypoxia and ischemia condition, IL-6 and TNF-α were associated with fetal behaviors and neuro-pathologies (47). Additionally, fetal immune response to the increased postnatal level of IFN-γ was associated with the degree of white matter damage in the brain (48). Therefore, our observation of the elevated cytokines in COVID-19 placentas could provide novel insights into the link with a higher rate of abnormal DDST in infants.

The baseline and laboratory characteristics of COVID-19 pregnant women were consistent with previous studies (2, 49, 50). In our study, there are 5 pregnant women infected with hepatitis B. We collected the results of second liver two half-and-half detect and HBV-DNA from medical records, and found that the HBV-DNA copy number of all 5 pregnant women were under 1 × 10^3^ U/mL. And theses pregnant women were recommended to the HBV mother-to-child blocking clinic of our hospital. None of the newborns were infected with HBV at birth. Previous studies have found that pregnant women infected with HBV increased the infant's risk of infection (51, 52). But there did not appear to be any reported effects on the infant's physical or neurodevelopment. Our follow-up results also suggested that the physical development of the infants delivered by these five pregnant women was normal and there was no DDST abnormality. A large-scale systematic review and meta-analysis showed that low birthweight and preterm birth were more probable in pregnant woman with COVID-19, and low birthweight was one of the most prevalent infant complications (50). In our study, the average birthweight of infants was slightly lower in the COVID-19 group, the median and rate of abnormal TNI in newborns were also significantly increased in the COVID-19 group. Previous studies suggested that TNI can be used not only as a marker for neonatal myocardial injury, but also a marker for neonatal hypoxic ischemic encephalopathy (53, 54). Our findings indicated that neonates born to COVID-19 mothers might be at risk for intrauterine ischemia and hypoxia.

In addition, the copy number of placental mtDNA has been functioned as a potential biomarker for placental insufficiency and intrauterine growth restriction (55–57). The placental mitochondrial DNA copy number has been associated with reduced birthweight in women with placental malaria (58). Here, we found that the placental mtDNA content was associated with GAD and birthweight. Consistently, the placental mtDNA content of preterm delivery was also higher than term delivery in COVID-19 pregnant women, reflecting the trend of different incidence of preterm delivery between the two groups. Furthermore, the placental mtDNA was positively correlated with TNI levels in newborns. Taken together, these findings indicated that the elevated placental mtDNA in COVID-19 was compensating for infant nutrient and oxygen requirements due to poor maternal vascular perfusion.

The three infants with abnormal DDST at 1–2 months of age passed the examinations later, supporting that neurodevelopmental delay might be short-term effects which could be corrected. Moreover, the placental mtDNA content of infants with abnormal DDST was increased compared to the mean of all infants from COVID-19 mothers. A previous study has shown that the placental mtDNA content was associated with performance of intelligence quotient in childhood, emphasizing the importance of the intrauterine environment for intelligence and the role of placental mitochondrial functions (59). Overall, our findings revealed the significance of placental mitochondrial functions during fetal brain development *in utero*.

In this study, we reported a link between placental mitochondrial function and early physical and neurobehavioral development in infants delivered by COVID-19 mothers. Importantly, the relationship between placental mitochondria functions and infant development reported here was based on ~9 months follow-up, our on-going observations would enhance our understanding of any long-lasting consequences due to COVID-19. There are some limitations in the current study. Our study was based on a cohort of 31 COVID-19 pregnant women in Wuhan. The relatively small sample size may lead to a bias. Of course, we cannot exclude the effect of the time of DDST administration. Future studies with a larger study population and longer-term follow-up information will provide a clearer picture of the mitochondrial dysfunction and infant outcomes for affected infants.

## Conclusions

The MVM, increased cytokines, and mtDNA content of placentas in mothers with COVID-19 appear to be associated with transient early fine motor abnormalities in infants. Our findings suggest that infants born to mothers with COVID-19 need developmental screening test in the early life, and provide evidence that could guide the prenatal and postnatal considerations.

## Data Availability Statement

The raw data supporting the conclusions of this article will be made available by the authors, without undue reservation.

## Ethics Statement

The studies involving human participants were reviewed and approved by Medical Ethical Committee of Zhongnan Hospital of Wuhan University. Written informed consent to participate in this study was provided by the participants' legal guardian/next of kin.

## Author Contributions

WZ, S-ML, and YZ: concept and design, critical revision of the manuscript for important intellectual content, and supervision. H-YL, JG, TW, GY, DZ, PY, and XY: acquisition, analysis, or interpretation of data. H-YL, JG, and YC: performing experiments. CZ: fitting model. H-YL and JG: statistical analysis. H-YL, JG, CZ, WZ, S-ML, and YZ: drafting of the manuscript. YZ: obtained funding. All authors approved the final manuscript.

## Funding

This study was supported partially by Hubei Provincial Science and Technology Department Novel Coronavirus Pneumonia Emergency Science and Technology Project (Grant No. 2020FCA011), Wuhan Novel Coronavirus Pneumonia Emergency Science and Technology Tackling Key Project (Grant No. 2020020201010011), Health Commission of Hubei Province Scientific Research Project (Grant No. WJ2019C002).

## Conflict of Interest

The authors declare that the research was conducted in the absence of any commercial or financial relationships that could be construed as a potential conflict of interest.

## Publisher's Note

All claims expressed in this article are solely those of the authors and do not necessarily represent those of their affiliated organizations, or those of the publisher, the editors and the reviewers. Any product that may be evaluated in this article, or claim that may be made by its manufacturer, is not guaranteed or endorsed by the publisher.
