# Supplementary material for: Transient Early Fine Motor Abnormalities in Infants Born to COVID-19 Mothers Are Associated With Placental Hypoxia and Ischemia
Source: Front Pediatr. 2022 Jan 6;9:793561. doi: 10.3389/fped.2021.793561 (PMC8772397; doi:10.3389/fped.2021.793561)
Supplement: Supplementary file 1 [file Data_Sheet_1.docx]

**Table S1** **Laboratory findings of pregnant women**

|  | **Normal Range** | **COVID-19**  **(N = 31）** | **Pneumonia**  **(N = 18)** | **Control**  **(N = 49)** | ***P* ^a^** | ***FDR*** |
| --- | --- | --- | --- | --- | --- | --- |
| **Blood routine examination** | | |  |  |  |  |
| WBC count,×10^9^/L | 3.5 - 9.5 | 8.4(6.9, 9.6) | 8.8(7.9, 11.5) | 8.6(7.1, 9.5) | 0.02 | 0.18 |
| Neutrophil count, ×10^9^/L | 1.8 - 6.3 | 6.6(4.2, 7.6) | 6.9(6.0, 9.7) | 6.2(5.1, 7.2) | 0.01 | 0.10 |
| Lymphocyte count, ×10^9^/L | 1.1 - 3.2 | 1.3(1.0, 1.6) | 1.4(1.3, 1.5) | 1.4(1.2, 1.8) | 0.67 | 0.80 |
| Basophil count, ×10^9^/L | 0 - 0.06 | 0.02(0.01, 0.04) | 0.03(0.02, 0.03) | 0(0, 0.03) | 0.20 | 0.46 |
| Eosinophil count, ×10^9^/L | 0.02 - 0.52 | 0.01(0, 0.03) | 0.02(0.01, 0.04) | 0.04(0, 0.09) | 0.09 | 0.31 |
| Monocyte count, ×10^9^/L | 0.1 - 0.6 | 0.5(0.3, 0.7) | 0.5(0.4, 0.6) | 0.6(0.5, 0.7) | 0.05 | 0.26 |
| Platelet count, ×10^9^/L | 125 - 350 | 197(151, 224) | 207(172, 266) | 192(141, 235) | 0.24 | 0.47 |
| RBC, ×10^9^/L | 3.8 - 5.1 | 4.1(3.7, 4.2) | 3.8(3.6, 4.2) | 3.9(3.7, 4.1) | 0.60 | 0.78 |
| Hemoglobin, g/L | 115 - 150 | 123(113, 133) | 119(114, 136) | 121(113, 129) | 0.43 | 0.65 |
| **Coagulation function** | |  |  |  |  |  |
| Prothrombin time, s | 9.4 - 12.5 | 10.4(9.6, 10.8) | 10.5(10.1, 11.0) | 10.6(10.1, 11.1) | 0.39 | 0.62 |
| PTTA, % | 80 - 130 | 123(117, 140) | 119(112, 130) | 112(103, 126) | 0.003 | 0.09 |
| Thrombin time, s | 10.3 - 16.6 | 14.5(13.5, 14.9) | 13.7(13.4, 14.1) | 13.2(12.7, 14.1) | 3×10^-4^ | 0.02 |
| APTT, s | 25.1 - 36.5 | 27.9(26.6, 30.6) | 26.8(26.1, 28.2) | 26.9(25.4, 28.6) | 0.05 | 0.26 |
| D-dimer, ng/mL | 0 - 500 | 648(485, 1106) | 762(502, 1333) | 592(418, 774) | 0.21 | 0.47 |
| Fibrinogen, mg/dL | 238 - 498 | 445(425, 507) | 446(437, 503) | 447(392, 476) | 0.20 | 0.46 |
| INR | 0.85 - 1.15 | 0.96(0.88, 0.99) | 0.96(0.93, 1.01) | 0.97(0.93, 1.02) | 0.37 | 0.62 |
| **Liver function** | |  |  |  |  |  |
| ALT, U/L | 7 - 45 | 14(11, 21) | 8(7, 10) | 10(8, 14) | 0.27 | 0.49 |
| AST, U/L | 13 - 35 | 24(20, 33) | 17(13, 21) | 18(15, 22) | 0.22 | 0.47 |
| AST/ALT | 0.2 - 2.0 | 1.7(1.3, 2.1) | 2.1(1.8, 2.4) | 1.7(1.5, 2.0) | 0.09 | 0.31 |
| Total bilirubin, μmol/L | 5 - 21 | 9(8, 11) | 9 (8, 12) | 8(6, 9) | 0.01 | 0.10 |
| Direct bilirubin, μmol/L | 0 - 7 | 2(1, 4) | 2(1, 3) | 2(1, 3) | 0.03 | 0.21 |
| Total protein, g/L | 65 - 85 | 66(61, 71) | 70(66, 71) | 64(60, 68) | 0.03 | 0.21 |
| Albumin, g/L | 40 - 55 | 35 (32, 38) | 37(35, 38) | 35(32, 37) | 0.62 | 0.78 |
| Globulin, g/L | 20 - 30 | 31 (29, 33) | 31(29, 35) | 29 (26, 32) | 0.04 | 0.25 |
| Albumin/Globulin | 1.5 - 2.5 | 1.1(1.1, 1.2) | 1.2(1.0, 1.2) | 1.2(1.0, 1.4) | 0.45 | 0.65 |
| GGT, U/L | 8 - 57 | 16(10, 29) | 9(7, 17) | 11(8, 16) | 0.11 | 0.32 |
| Total bile acid, mmol/L | 0.85- 1.51 | 3.25(2.08, 6.20) | 1.95(1.18, 2.90) | 2.60(1.60, 3.75) | 0.96 | 0.96 |
| ALP, U/L | 30 - 120 | 229(175, 288) | 181(129, 246) | 189 (140, 228) | 0.08 | 0.31 |
| Glucose, mmol/L | 3.9 - 6.1 | 4.7(4.3, 5.7) | 4.4(4.1, 5.1) | 4.5(4.0, 5.2) | 0.07 | 0.31 |
| **Renal function** | |  |  |  |  |  |
| BUN, mmol/L | 2.8 - 7.6 | 3.0(1.9, 4.1) | 2.6(2.1, 3.7) | 3.4(2.8, 4.6) | 0.09 | 0.31 |
| CO_2_, mmol/L | 21 - 29 | 20(17, 23) | 19(18, 22) | 20(18, 22) | 0.76 | 0.83 |
| Creatinine, μmol/L | 64 - 104 | 53(46, 61) | 49(42, 55) | 50 (43, 56) | 0.10 | 0.32 |
| Cystatin C, mg/L | 0 - 1.2 | 1.2(1.0, 1.3) | 1.1(0.9, 1.3) | 1.0(0.9, 1.2) | 0.01 | 0.10 |
| Uric Acid, μmol/L | 208 - 428 | 334(296, 413) | 323(260, 366) | 326 (278, 392) | 0.31 | 0.55 |
| eGFR, mL/min/1·73m^2^ | > 90 | 127(122, 133) | 130 (117, 132) | 126 (115, 135) | 0.95 | 0.96 |
| β2-M, μg/L | 1000 - 3000 | 1587 (1237, 1828) | 1527(1189, 1689) | 1544(1363, 1816) | 0.68 | 0.80 |
| **Myocardial enzymes** | |  |  |  |  |  |
| CK, U/L | 0 - 171 | 37(27, 80) | 31(25, 61) | NA | 0.44 | 0.65 |
| CK-MB, ng/mL | 0 - 25 | 13(11, 18) | 9(8, 13) | NA | 0.25 | 0.47 |
| LDH, U/L | 125-243 | 198(173, 252) | 169(152, 208) | NA | 0.01 | 0.10 |
| LDH1, U/L | 15-65 | 33(30, 46) | 35(29, 40) | NA | 0.47 | 0.66 |
| **Serum electrolytes** | |  |  |  |  |  |
| Kalium, mmol/L | 3.5 - 5.3 | 4.1(3.9, 4.3) | 4.1(3.9, 4.2) | 4.0(3.8, 4.2) | 0.24 | 0.47 |
| Sodium, mmol/L | 137 - 147 | 137(136, 138) | 136(135, 137) | 136 (135, 138) | 0.39 | 0.62 |
| Calcium, mmol/L | 2.11 - 2.52 | 2.26(2.15, 2.35) | 2.27(2.20, 2.35) | 2.24(2.14, 2.28) | 0.06 | 0.29 |
| Chlorine, mmol/L | 99 - 110 | 105(103, 107) | 104(101, 106) | 105(103, 106) | 0.11 | 0.32 |
| Magnesium, mmol/L | 0.85 - 1.15 | 0.92(0.85, 0.98) | 0.92(0.80, 1.00) | 0.91(0.84, 0.99) | 0.16 | 0.44 |
| Phosphate, mmol/L | 0.85 - 1.51 | 1.18(1.04, 1.26) | 1.15(1.06, 1.34) | 1.19(1.07, 1.29) | 0.72 | 0.81 |
| **Inflammatory markers** | | |  |  |  |  |
| CRP, mg/L | 0 - 10 | 6(3, 16) | 4(3, 6) | NA | 0.18 | 0.45 |
| ESR, mm/h | 0-20 | 36(21, 48) | 31(27, 44) | NA | 0.83 | 0.87 |
| IL-6, pg/mL | 0 - 7 | 7(5, 10) | 6(4, 9) | NA | 0.76 | 0.83 |
| Amyloid protein A, mg/L | 0 - 10 | 10(6, 20) | 8(5, 15) | NA | 0.45 | 0.65 |
| C1q, mg/L | 159 - 233 | 181(152, 220) | 185 (163, 208) | NA | 0.82 | 0.87 |
| Cytokine B, g/L | 100 - 400 | 377(333, 399) | 336 (322, 373) | NA | 0.50 | 0.67 |
| **Blood lymphocyte count** | | |  |  |  |  |
| CD3^+^ T，% | 38.56-70.06 | 75.44(73.07, 79.93) | 78.59(72.02, 83.88) | NA | 0.68 | 0.80 |
| CD3^+^ T count, cells/μL | 805-4459 | 1156(852, 1474) | 1028(809, 1212) | NA | 0.17 | 0.44 |
| CD3^+^CD4^+^Th, % | 14.21-36.99 | 39.78(35.56, 45.52) | 41.33(34.29, 45.33) | NA | 0.70 | 0.8 |
| CD3^+^CD4^+^Th count, cells/μL | 345-2350 | 575(458, 834) | 558(423, 627) | NA | 0.17 | 0.44 |
| CD3^+^CD8^+^ Ts, % | 13.24-38.53 | 33.57(24.25, 37.81) | 35.38(29.24, 37.49) | NA | 0.58 | 0.77 |
| CD3^+^CD8^+^ Ts count, cells/μL | 345-2350 | 497(336, 667) | 436(372, 511) | NA | 0.24 | 0.47 |
| CD19^+^ B, % | 10.86-28.03 | 10.88(7.97, 12.06) | 9.07(7.50, 14.52) | NA | 0.93 | 0.96 |
| CD19^+^ B count, cells/μL | 240-1317 | 139(115, 214) | 129(93, 180) | NA | 0.37 | 0.62 |
| CD16^+^CD56^+^NK, % | 7.92-33.99 | 12.84(7.90, 16.57) | 10.49(7.29, 15.92) | NA | 0.66 | 0.8 |
| CD16^+^CD56^+^NK count, cells/μL | 210-1514 | 190(133, 256) | 134(91, 189) | NA | 0.48 | 0.66 |

a, *P*-values were derived from one-way ANOVA analysis, indicating differences across COVID-19, non-COVID-19 pneumonia and control pregnant women. *P*-value < 0.05 was considered statistically significant. FDR, *P* values were adjusted for multiple comparisons to control the false-discovery rate. WBC, white blood cell; RBC, red blood cell; ESR, erythrocyte sedimentation rate; PTTA, prothrombin time activity; APTT, activated partial thromboplastin time; INR, international normalized ratio; ALT, alanine aminotransaminase; AST, aspartate aminotransferase; GGT, gamma-glutamyl transpeptidase; ALP; Alkaline phosphatase BUN, blood urea nitrogen; eGFR, estimated glomerular filtration rate; Beta-2-microglobulin, β_2_-M; LDH, lactate dehydrogenase; CK, Creatine kinase; CK-MB, Creatine kinase-MB; LDH1, LDH isoenzyme-1; HBDH, alpha-hydroxybutyrate dehydrogenase; CRP, C-reactive protein; IL-6, interleukin-6; C1q, Complement component 1q; CD3^+^ T, CD3^+^ T lymphocytes；CD3^+^CD4^+^ Th, CD3^+^CD4^+^ T helper lymphocytes; CD3^+^CD8^+^ Ts, CD3^+^CD8^+^ T suppressor lymphocytes; CD16^+^CD56^+^NK, CD16^+^CD56^+^ Natural killer cell.

**Table S2** **Abnormalities of** **laboratory findings of pregnant women**

|  | **NO. (%)** |  |  |  |
| --- | --- | --- | --- | --- |
|  | **COVID-19** | **Pneumonia** | ***P* ^a^** | ***FDR*** |
| **Myocardial enzymes** | |  |  |  |
| CK, U/L | 0/24(0) | 0/20(0) | NA | NA |
| CK-MB, ng/mL | 3/25(12.0) | 0/19(0) | 0.25 | 0.83 |
| LDH, U/L | 7/25(28.0) | 0/19(0) | 0.01 | 0.20 |
| LDH1, U/L | 1/15(6.7) | 0/10(0) | >0.99 | NA |
| **Inflammatory markers** |  |  |  |  |
| CRP, mg/L | 9/27(33.3) | 5/27(18.5) | 0.35 | 0.88 |
| ESR, mm/h | 16/20(80.0) | 19/20(95.0) | 0.34 | 0.88 |
| IL-6, pg/mL | 9/20(45.0) | 11/21(52.4) | 0.64 | 1.00 |
| Amyloid protein A, mg/L | 9/18(50.0) | 5/17(29.4) | 0.21 | 0.83 |
| C1q, mg/L | 8/19(42.1) | 6/26(23.1) | 0.17 | 0.83 |
| Cytokine B, g/L | 3/14(21.4) | 2/17(11.8) | 0.64 | 1.00 |
| **Blood lymphocyte count** |  |  |  |  |
| CD3^+^ T，% | 17/21(81.0) | 14/17(82.4) | >0.99 | NA |
| CD3^+^ T count, cells/μL | 4/21(19.1) | 4/17(23.5) | >0.99 | NA |
| CD3^+^CD4^+^Th, % | 15/21(71.4) | 11/17(64.7) | 0.66 | 1.00 |
| CD3^+^CD4^+^Th count, cells/μL | 0/21(0) | 3/17(17.7) | 0.08 | 0.80 |
| CD3^+^CD8^+^ Ts, % | 5/21(23.8) | 4/17(23.5) | >0.99 | NA |
| CD3^+^CD8^+^ Ts count, cells/μL | 5/21(23.8) | 4/17(23.5) | >0.99 | NA |
| CD19^+^ B, % | 10/21(47.6) | 9/17(52.9) | 0.74 | 1.00 |
| CD19^+^ B count, cells/μL | 18/21(85.7) | 15/17(88.2) | >0.99 | NA |
| CD16^+^CD56^+^NK, % | 4/21(19.1) | 5/17(29.4) | 0.70 | 1.00 |
| CD16^+^CD56^+^NK count, cells/μL | 13/21(61.9) | 15/17(88.2) | 0.14 | 0.83 |

a, *P*-values were derived from one-way ANOVA analysis, indicating differences across COVID-19, non-COVID-19 pneumonia and control pregnant women. *P*-value < 0.05 was considered statistically significant. FDR, *P* values were adjusted for multiple comparisons to control the false-discovery rate. WBC, white blood cell; RBC, red blood cell; ESR, erythrocyte sedimentation rate; PTTA, prothrombin time activity; APTT, activated partial thromboplastin time; INR, international normalized ratio; ALT, alanine aminotransaminase; AST, aspartate aminotransferase; GGT, gamma-glutamyl transpeptidase; ALP; Alkaline phosphatase BUN, blood urea nitrogen; eGFR, estimated glomerular filtration rate; Beta-2-microglobulin, β_2_-M; LDH, lactate dehydrogenase; CK, Creatine kinase; CK-MB, Creatine kinase-MB; LDH1, LDH isoenzyme-1; HBDH, alpha-hydroxybutyrate dehydrogenase; CRP, C-reactive protein; IL-6, interleukin-6; C1q, Complement component 1q; CD3^+^ T, CD3^+^ T lymphocytes；CD3^+^CD4^+^ Th, CD3^+^CD4^+^ T helper lymphocytes; CD3^+^CD8^+^ Ts, CD3^+^CD8^+^ T suppress lymphocytes; CD16^+^CD56^+^NK, CD16^+^CD56^+^ Natural killer cell.

**Table S3** **Laboratory findings of newborns**

|  |  | **COVID-19**  **(N = 33)** | **non-COVID-19**  **(N = 39)** | ***P* ^a^** | ***FDR*** |
| --- | --- | --- | --- | --- | --- |
| **Normal Range** | |  |  |  |  |
| **Blood routine examination** | |  |  |  |  |
| WBC count, ×10^9^/L | 5 - 30 | 13(9, 15) | 15 (13, 20) | 0.02 | 0.11 |
| Neutrophil count, ×10^9^/L | 3.9 - 9.4 | 7.4(4.2, 9.2) | 7.9(5.2, 12.0) | 0.99 | 1.00 |
| Lymphocyte count, ×10^9^/L | 1.15 – 17.00 | 3.59(2.69, 4.66) | 5.36(3.92, 6.98) | 0.005 | 0.06 |
| Monocyte count, ×10^9^/L | 0.2 - 3.1 | 1.0(0.7, 1.5) | 1.4(0.9, 1.8) | 0.55 | 0.80 |
| Platelet count, ×10^9^/L | 242 - 378 | 282(246, 331) | 323(222, 378) | 0.36 | 0.64 |
| **Coagulation function** |  |  |  |  |  |
| Prothrombin time, s | 13 - 20 | 15(14, 17) | 15(15, 19) | 0.2 | 0.40 |
| APTT, s | 45 - 65 | 52(45, 59) | 46 (40, 62) | 0.54 | 0.80 |
| **Liver/Renal function** |  |  |  |  |  |
| ALT, U/L | 6 - 40 | 10(9, 12) | 13(8, 15) | 0.03 | 0.12 |
| AST, U/L | 3-100 | 42(38, 55) | 45(34, 52) | 0.81 | 1.00 |
| Total bilirubin, μmol/L | term newborns <204;  premature <255 | 39(33, 45) | 42(36, 49) | 0.89 | 1.00 |
| BUN, mmol/L | 2.8 - 7.9 | 3.0(2.1, 3.5) | 3.3 (3.0, 3.7) | 0.12 | 0.32 |
| Creatinine, μmol/L | 64 - 104 | 59(49, 64) | 59(51, 66) | 0.62 | 0.83 |
| **Myocardial enzymes** |  |  |  |  |  |
| TNI, pg/mL | 0 - 26.2 | 15.0(6.6, 24.7) | 7.9(5.0, 14.2) | 0.007 | 0.05 |
| CK-MB, ng/mL | 2.3 - 60.0 | 8.1(5. 8, 11.6) | 8.4(6.5, 12.3) | 0.17 | 0.39 |
| **Inflammatory markers** |  |  |  |  |  |
| IL-6, pg/mL | < 7.00 | 6.69(2.22, 17.38) | 4.03(2.51, 8.62) | 0.12 | 0.32 |

a, *P*-values were derived from student’s t test, indicating differences between infants born to COVID-19 and non-COVID-19 pregnant women. *P*-value < 0.05 was considered statistically significant. FDR, *P* values were adjusted for multiple comparisons to control the false-discovery rate. WBC, white blood cell; RBC, red blood cell; PTTA, prothrombin time activity; APTT, activated partial thromboplastin time; ALT, alanine aminotransaminase; AST, aspartate aminotransferase; BUN, blood urea nitrogen; TNI, troponin Ⅰ; CK-MB, Creatine kinase-MB; IL-6, interleukin-6.

**Table S4 Abnormalities of laboratory findings in newborns**

|  | **NO. (%)** |  |  |
| --- | --- | --- | --- |
|  | **COVID-19** | **non-COVID-19** | ***P* ^a^** |
|  | **(N = 33)** | **(N = 39)** |  |
| **Blood routine examination** | | |  |
| WBC count, ×10^9^/L | 0/27(0) | 0/10(0) | >0.99 |
| Neutrophil count, ×10^9^/L | 5/27(18.5) | 3/10(30.0) | 0.66 |
| Lymphocyte count, ×10^9^/L | 2/27(7.4) | 0/10(0) | >0.99 |
| Monocyte count, ×10^9^/L | 1/25(4.0) | 0/10(0) | >0.99 |
| Platelet count, ×10^9^/L | 2/26(7.7) | 2/10(20.0) | 0.31 |
| **Coagulation function** | |  |  |
| Prothrombin time, s | 2/17(11.8) | 1/9(11.1) | >0.99 |
| APTT, s | 5/17(29.4) | 5/9(55.6) | 0.23 |
| **Liver/Renal function** | |  |  |
| ALT, U/L | 2/25(8.0) | 2/23(8.7) | >0.99 |
| AST, U/L | 0/25(0) | 1/23(4.4) | 0.48 |
| ,tal bilirubin, μmol/L | 0/25(0) | 0/23(0) | >0.99 |
| BUN, mmol/L | 0/25(0) | 0/10(0) | >0.99 |
| Creatinine, μmol/L | 0/25(0) | 0/10(0) | >0.99 |
| **Myocardial enzymes** | |  |  |
| TNI, pg/mL | 5/25(20) | 0/23(0) | 0.05 |
| CK-MB, ng/mL | 1/26(3.9) | 1/23(4.4) | >0.99 |
| **Inflamma,ry markers** | |  |  |
| IL-6, pg/mL | 11/24(45.8) | 8/23(34.8) | 0.44 |

a, *P*-values were derived from chi-square test, indicating differences between infants born , COVID-19 and non-COVID-19 pregnant women. *P*-value < 0.05 was considered statistically significant. WBC, white blood cell; RBC, red blood cell; PTTA, prothrombin time activity; APTT, activated partial thromboplastin time; ALT, alanine aminotransaminase; AST, aspartate aminotransferase; BUN, blood urea nitrogen; TNI, troponin Ⅰ; CK-MB, Creatine kinase-MB; IL-6, interleukin-6.

**Table S5 Baseline characteristics and clinical characteristics of the follow-up infants**

| **Variable** | **COVID-19**  **(N = 28)** | **non-COVID-19**  **(N = 48)** | ***P* ^a^** |
| --- | --- | --- | --- |
|  |  |  |  |
| Male ^b^ | 16 (57.1) | 33 (67.3) | 0.46 |
| Maternal age, years | 30.6±3.3 | 33.0±4.8 | 0.07 |
| GAD, weeks | 38.0±0.4 | 38.4±0.3 | 0.47 |
| Birthweight, kg | 2.94±1.28 | 3.18±0.08 | 0.24 |
| Body length, cm | 48.46±0.78 | 48.64±0.93 | 0.90 |
| Head circumference, cm | 33.64±0.5 | 33.68±0.30 | 0.95 |
| Premature delivery ^b^ | 5(17.9) | 7(14.3) | 0.75 |

a, *P*-values were derived from student’s test (quantitative variables) or chi-square test (categorical variables) indicating differences between infants of COVID-19 and non-COVID-19 pregnant women. b, data are expressed as percentage. *P*-value < 0.05 was considered statistically significant. GAD, Gestational age at delivery.

**Table S6 Characteristics of follow-up COVID-19 mothers and infants**

| **Case** | **Mothers** | | | |  |  | **Infants** | | | | |
| --- | --- | --- | --- | --- | --- | --- | --- | --- | --- | --- | --- |
|  | SARS-CoV-2 nucleic acid | SARS-CoV-2 antibody | Age  (years) | Comorbidities | mtDNA content |  | Sex | GAD  (weeks) | Birth weight  (kg) | DDST | TNI  (pg/ml) |
|  |  |  |  |  |  |  |  |  |  |  |  |
| **1** | + | + | 27 | No | 1.12 |  | Male | 39.29 | 3.15 | Normal | 3.1 |
| **2** | + | NA | 32 | Hepatitis B virus carrier；PROM | 1.15 |  | Male | 37.57 | 2.95 | Normal | 3.0 |
| **3** | + | NA | 30 | GDM、PIH | NA |  | Male | 37.43 | 3.21 | Normal | 57.1 |
| **4** | + | + | 33 | Influenza infection | 1.13 |  | Male | 37.29 | 2.09 | Normal | NA |
| **5** | + | + | 26 | Severe preeclampsia | 1.11 |  | Female | 36.43 | 1.09 | Normal | NA |
| **6** | + | NA | 40 | PIH | 1.21 |  | Male | 36.00 | 3.80 | Normal | NA |
| **7** | + | + | 27 | No | 1.11 |  | Male | 38.43 | 3.73 | Normal | NA |
| **8** | - | + | 35 | No | 1.06 |  | Female | 39.00 | 2.87 | Normal | 13.9 |
| **9** | + | - | 33 | No | 1.04 |  | Male | 41.29 | 3.94 | Normal | 6.8 |
| **10** | - | + | 38 | PROM | 1.17 |  | Female | 38.00 | 2.77 | Questionable | 7.6 |
| **11** | - | + | 27 | PROM | 0.88 |  | Male | 38.43 | 2.89 | Normal | 14.2 |
| **12** | - | + | 33 | PIH | 1.21 |  | Female | 38.29 | 3.79 | Questionable | 67.5 |
| **13** | - | + | 32 | Placenta praevia;  Test-tube baby | 1.24 |  | Female | 37.57 | 2.63 | Questionable | 44.9 |
| **14** | - | + | 29 | No | 1.14 |  | Male | 39.43 | 3.12 | Normal | 23.4 |
| **15** | - | + | 30 | No | 1.12 |  | Female | 40.29 | 3.12 | Normal | 16.7 |
| **16** | + | + | 27 | Severe preeclampsia; Heart failure | 1.15 |  | Male | 32.00 | 1.50 | Normal | 5.7 |
| **17** | + | + | 30 | Influenza infection | 1.21 |  | Male | 38.86 | 2.85 | Normal | 12.4 |
| **18** | - | + | 30 | Hepatitis B virus carrier；Placental abruption | 1.29 |  | Female | 31.86 | 1.57 | Normal | 25.3 |
| **19** | - | + | 30 | Hepatitis B virus carrier；Placental abruption | 1.29 |  | Female | 31.86 | 1.61 | Normal | 23.5 |
| **20** | + | + | 29 | No | 1.18 |  | Male | 39.14 | 3.85 | Normal | NA |
| **21** | + | NA | 28 | No | NA |  | Female | 40.29 | 3.59 | Normal | NA |
| **22** | + | NA | 31 | Twin pregnancy、PIH | NA |  | Male | 37.14 | 2.81 | Questionable | 6.3 |
| **23** | + | NA | 31 | Twin pregnancy、PIH | NA |  | Female | 37.14 | 2.45 | Normal | 9.2 |
| **24** | + | NA | 29 | Hepatitis B virus carrier；PIH；Influenza infection | NA |  | Female | 40.43 | 3.66 | Normal | 40.1 |
| **25** | - | + | 27 | No | NA |  | Female | 38.71 | 3.11 | Normal | 4.5 |
| **26** | - | + | 31 | No | NA |  | Male | 39.43 | 2.57 | Questionable | 24.0 |
| **27** | - | + | 32 | PROM | NA |  | Male | 39.14 | 3.07 | Normal | 35.6 |
| **28** | - | + | 34 | GDM | NA |  | Male | 39.57 | 2.82 | Normal | 19.7 |

The red color fonts indicate the information of abnormal infants. GAD, Gestational age at delivery; DDST, Denver Developmental Screening Test; TNI, cardiac troponin Ⅰ; −, negative; +, positive; PROM, premature rupture of membrane; GDM, Gestational diabetes mellitus; PIH, Pregnancy-induced hypertension

**Table S7** **Summary of clinical and pathological findings**

| **Case** | **Symptoms of onset** | **Gestation at diagnosis (weeks)** | **Gestation at delivery (weeks)** | **Obstetric complications** | **APGAR score at 1minute/5 minutes** | **Neonatal weight (kg)** | **Placental**  **pathology** | **Control placentas: week/diagnosis** |
| --- | --- | --- | --- | --- | --- | --- | --- | --- |
| **1** | Fever,  Cough | 35 | 39 | MSAF | 9/10 | 2.85 | MVM  Lymphocyte infiltration,  Intervillous fibrin calcification,  STB knot,  Calcification, villi infarction,  Subchorionic hematoma,  charangoists in villi | C1:39/ Eutocia  C2:38/ Eutocia  C3: 39/ Eutocia |
| **2** | Fever | 37 | 38 | Oligohydramnios | 9/10 | 2.80 | MVM,  Distal villous hypoplasia,  Lymphocyte infiltration,  Intervillous fibrin,  Calcification, villi infarction,  STB Knot | C1:39/ Eutocia  C2:38/ Eutocia  C3: 39/ Eutocia |
| **3** | Fever | 38 | 39 | None | 9/10 | 3.15 | Sub conjunctivitis,  Lymphocyte infiltration | C1:39/ Eutocia |
| **4** | Fever,  cough | 38 | 39^+4^ | PROM,  MSAF | 8/10 | 3.53 | MVM,  Lymphocyte infiltration,  Intervillous fibrin,  villi infarction | C4:39/PROM  C7;36/PROM,  PTB |
| **5** | Fever, | 31 | 32 | PROM,  Multiple pregnancy,  placental abruption | 8/8  8/9 | 1.57/1.61 | MVM,  Edema in a few villi,  Accelerated villous maturation,  Calcification, villi infarction,  Subchorionic hematoma | C5:32/PROM,  PTB |
| **6** | cough | 37 | 38^+3^ | PROM, oligohydramnios | 9/10 | 2.89 | Lymphocyte infiltration,  intervillous fibrin, | C6:38/PROM  C7;36/PROM,  PTB |
| **7** | Fever,  cough | 35 | 36^+2^ | PROM | 9/10 | 2.64 | lymphocyte infiltration,  Intervillous fibrin,  Calcification  STB Knot | C6:38/PROM  C7:36/PROM,  PTB |
| **8** | Fever,  Cough | 34 | 38^+3^ | PROM,  MSAF | 8/10 | 3.07 | fibrin, villi infarction,  Calcification  STB Knot,  Charangoists in villi, | C6:38/PROM  C7:36/PROM,  PTB  C8:38/PROM |
| **9** | Cough | 36^+3^ | 38^+2^ | PIH | 9/10 | 3.79 | MVM,  lymphocyte infiltration,  fibrin, villi infarction  Calcification | C9:38/PIH  C10:32/PIH,  PTB |
| **10** | Fever,  Cough | 28^+4^ | 32 | EOSP  Fetal distress | 7/8 | 1.50 | MVM,  Calcification,  Fibrin, villi infarction,  STB Knot,  Subchorionic hematoma,  Distal villous hypoplasia, charangoists in villi | C9:38/PIH  C10:32/PIH,  PTB |

PROM: premature rupture of membrane, IUGR: intrauterine growth retardation, MSAF: meconium staining of amniotic fluid, PIH: pregnancy-induced hypertension, EOSP: Early onset of severe preeclampsia, MVM: maternal vascular malperfusion, PTB: pre-term birth, STB: syncytiotrophoblast.

**Table S8 The placental** **histological features of H&E staining**

| **Histological features** | **COVID-19** | **non-COVID-19** | ***P* Value ^a^** |
| --- | --- | --- | --- |
|  | **(N = 10)** | **(N = 10)** |  |
| Decidual arteriopathy | 6(60) | 1(10) | 0.06 |
| Increased syncytial knots | 5(50) | 2(20) | 0.35 |
| Increased intervillous fibrin | 7(70) | 0(0) | 0.03 |
| Intervillous thrombus | 4(40) | 1(0) | 0.30 |
| Villous infarction | 6(60) | 2(20) | 0.17 |

a, *P*-values were derived from chi-square test (categorical variables) indicating differences between Covid-19 and non-COVID-19 pregnant women. *P* value < 0.05 was considered statistically significant.
